# Resveratrol Production in Yeast Hosts: Current Status and Perspectives

**DOI:** 10.3390/biom11060830

**Published:** 2021-06-02

**Authors:** Gehad G. Ibrahim, Jinyong Yan, Li Xu, Min Yang, Yunjun Yan

**Affiliations:** 1 Key Laboratory of Molecular Biophysics of the Ministry of Education, College of Life Science and Technology, Huazhong University of Science and Technology, Wuhan 430074, China; ggibrahim@zu.edu.eg (G.G.I.); yjiny@126.com (J.Y.); xuli@mail.hust.edu.cn (L.X.); 2020010082@mail.hust.edu.cn (M.Y.); 2 Department of Genetics, Faculty of Agriculture, Zagazig University, Zagazig 44511, Egypt

**Keywords:** resveratrol, yeast hosts, pathway engineering, metabolic engineering

## Abstract

Resveratrol is a plant secondary metabolite known for its therapeutic applications as an antioxidant, anti-cancer, anti-inflammatory, anti-aging, cardio-protective, and neuroprotective agent. Topical formulas of resveratrol are also used for skin disease management and in cosmetic industries. Due to its importance, high resveratrol production is urgently required. Since the last decade, intensive efforts have been devoted to obtaining resveratrol from microorganisms by pathway and metabolic engineering. Yeasts were proven to be excellent host candidates for resveratrol production. In addition to the similar intracellular compartments between yeasts and plants, yeasts exhibit the ability to express genes coding for plant-derived enzymes and to perform post-translational modification. Therefore, this review summarizes the attempts to use yeasts as a platform for resveratrol synthesis as the next promising route in producing high titers of resveratrol from genetically engineered strains.

## 1. Introduction

**Figure 1:**
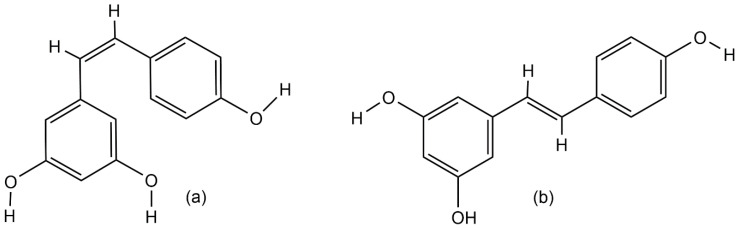
Chemical structures of resveratrol: (**a**) *cis*-resveratrol isomer and (**b**) *trans*-resveratrol isomer.

Resveratrol (3,4′,5-trihydroxystilbene) is a natural polyphenolic phytoalexin that belongs to the stilbenoids group of secondary metabolites [1,2,3]. Stilbenes are known for their ability to protect plants from UV light and the effect of chemical fertilizers [4], and for the defense of plants against biotic stresses such as bacterial, fungal, or nematode infections [3,5]. Resveratrol was first identified in 1940 as a constitutive compound of white hellebore roots (*Veratrum grandiflorum*) [6]. The richest source of resveratrol was found in *Polygonum cuspidatum* roots, the extract of which is widely used in traditional Chinese and Japanese medicine to treat human fungal diseases, such as gonococcal infection, suppurative dermatitis, tinea favosa and tinea pedis infections, hyperlipidemia, arteriosclerosis, and inflammations [1,7]. Thus far, resveratrol has been found in various plant species, such as grape berries [8], blueberries, cranberries (*Vaccinium* spp.) [9,10], blackberries, mulberries (*Rubus* and *Morus* spp.) [11,12], peanuts (*Arachis hypogaea*) [13], and jackfruit (*Artocarpus heterophyllus*) [11]. Naturally, there are two isomeric forms of resveratrol (Figure 1) but the *trans* isomer is the biologically active form [4]. However, under high pH values or UV-light exposure, resveratrol converts from the *trans* isoform into the *cis* isomer [14]. Both isomers exist as glucosides, and 3-*O*-β-d-resveratrol glucoside (piceid or polydatin) is the richest source of resveratrol in the glucoside form [1,14,15].

Resveratrol was found to be the reason for the well-known French Paradox, assuming that, despite the high consumption of saturated fats, French people show lower coronary heart disease incidence than other communities. This phenomenon can be explained by the consumption of red wine, which is a source of resveratrol [16,17]. 

Resveratrol has been widely studied in the past three decades [18,19] due to the accumulated evidence of its therapeutic properties. Resveratrol exhibits biological activities as a cardioprotective [20], anti-cancer [21,22,23,24], and possible anti-inflammatory [25] agent. Additional clinical evidence points to resveratrol’s role in cardiovascular disorder treatment [26], its impact on cell immunomodulation [27], and its role in lowering blood pressure [28]. Resveratrol has been suggested to have protective effects on neurodegenerative diseases, such as Alzheimer’s and Parkinson’s diseases [29,30,31,32]. Furthermore, resveratrol exhibits a positive role in treating periodontitis-related tissue defects and increased bone formation in mice morals [33].

In recent years, resveratrol’s topical formulations have been used in cosmetic skincare products [34] and skin disease management. Topical formulations of resveratrol have been reported to be valuable in treating skin diseases, such as acne, eczema, exfoliation, and psoriasis [35,36], and as a potent whitening agent [1]. Resveratrol’s antioxidant, antimicrobial, and antiviral effects provide skin protection from infections and ultraviolet-radiation-mediated oxidative stress (resulting in skin cancer and actinic keratosis) and show anti-aging properties [16,37]. Resveratrol was also found to accelerate skin wound healing [38].

Similar to glucosylated and methylated resveratrol derivatives, resveratrol oligomers present beneficial biological activities. Resveratrol-3-*O*-d-glycoside (piceid) shows antioxidant, anti-inflammatory, antitumor, hepatoprotective, and neuroprotective effects [39]. Many methylated derivatives, such as pterostilbene, trimethoxystilbene, and DMU212, possess more desirable pharmacokinetic properties than resveratrol and exhibit cardioprotective [40], anti-cancer [41,42,43], and neuroprotective activities [44,45]. Similarly, resveratrol oligomers such as pallidol [46,47], ε-viniferin [46,48,49,50], and labruscol [51] exhibit cytotoxic effects on cancer cell lines. Some isolated polymerized stilbenes, such as hopeaphenol, were found to inhibit the viability of cancer cells [52].

## 2. Resveratrol Biosynthesis in Nature

**Figure 2:**
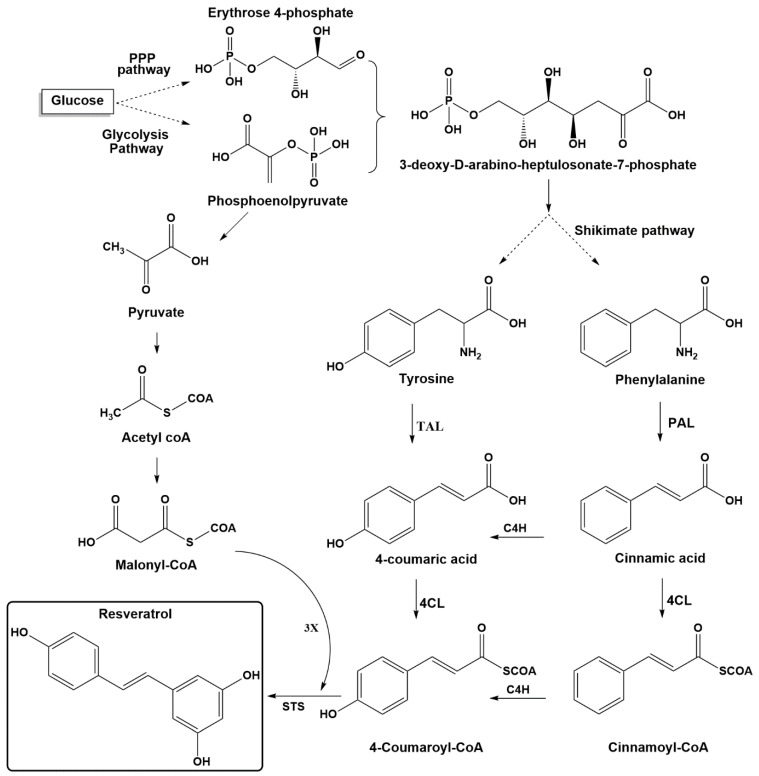
Resveratrol biosynthesis via the phenylalanine/tyrosine pathway. PPP: pentose phosphate pathway, TAL: tyrosine ammonia-lyase, PAL: phenylalanine ammonia lyase, C4H: cinnamate 4-hydroxylase, 4CL: 4-coumaroyl-coA ligase, STS: stilbene synthase.

Stilbenes are plant secondary metabolites built from joining two aromatic rings with an ethylene bridge (1,2-diphenylethylene backbone) to form the basic C6-C2-C6 skeleton. Stilbenes share many similarities in their structure, biosynthesis, and biological activities with phenylpropanoids and flavonoids, which originate from the same pathway. The key components for biosynthesis of stilbenes are malonyl-CoA, phenylalanine, and tyrosine (Figure 2). In glycolysis pathway, glucose is converted into pyruvic acid, which leads to acetyl coenzyme A. The latter is the precursor of malonyl-CoA, which plays an essential role in biosynthesis pathways for many secondary plant compounds. The condensation of erythrose-4-phosphate (four carbons), which is generated from the pentose phosphate pathway (parallel reaction pathway to glycolysis) and phosphoenol pyruvate (three carbons, an intermediate in glycolysis), yields the seven-carbon shikimate skeleton. The latter compound is then used in the shikimic acid pathway to generate the aromatic amino acids phenylalanine and tyrosine [1,53].

The biosynthesis pathway for resveratrol starts with phenylalanine or tyrosine, as shown in Figure 2. The first step is to obtain *para*-coumaric acid from both pathways. The phenylalanine ammonia-lyase (PAL) transforms phenylalanine into cinnamic acid, which is further processed to generate 4-coumaric acid (*para*-coumaric acid) using cinnamate-4-hydroxylase (C4H). Tyrosine ammonia-lyase (TAL) can directly generate *p*-coumaric acid from tyrosine. The second step includes transformation of *para*-coumaric acid into *para*-coumaroyl-CoA by *para*-coumaroyl coenzyme A ligase (4CL). The final step involves the condensation of three units of malonyl-CoA with *p*-coumaroyl-CoA through stilbene synthase (STS) [1,19,54].

The STS genes are usually expressed when induced by biotic or abiotic stimuli [55]. Although STS genes are limited to a few plant families, the *Cyperaceae*, *Dipterocarpaceae*, *Fabaceae*, *Gnetaceae*, *Pinaceae*, and *Vitaceae* families have been reported to have a high degree of STS expression [56,57]. Sequencing of the genome of *Vitis vinifera* led to a broad diversification of the STS genes; at least 33 full-length coding genes were identified [58], which suggests complex regulation pathways, including the action of various transcription factors such as MYB and WRKY [59,60,61].

Starting from resveratrol, more modifications can be accomplished on its structure by various decorating enzymes. Many stilbene derivatives possess antioxidant and antifungal activities after methylation [62] or glucosylation of the aromatic hydroxyl groups [63]. Polyphenolic secondary metabolites can also be obtained from the oxidation of resveratrol by peroxidases and laccases, allowing for 2−8 resveratrol molecules to condensate [55]. 

## 3. Resveratrol Production by Transgenic Yeasts

Due to resveratrol’s pharmacological importance and its possible health and disease applications, resveratrol large-scale production has become a necessity. In nature, plants are the resource for resveratrol production; however, their production is limited by high costs; low quantities (the highest resveratrol concentration in plants was found in the seeds of *Paeonia suffruticosa* Andr. var. *papaveracea* (Andr.) Kerner with titers of 0.87 g/kg) [64]; lack of plant sources; and difficulties in the extraction, purification, and concentration processes. Transgenic plants and plant cell suspensions are two methods used for resveratrol production. Although engineered plants are usually restricted to enhancing the expression of STS genes and they have high productivity (up to 650 mg/kg FW of resveratrol), the use of elicitors, long production times, purity, and engineering process difficulties compared with microorganisms, remain as drawbacks for using this method [65]. Similar to transgenic plants, plant cell suspensions need elicitors to induce cells to produce resveratrol with resveratrol titer up to 5 g/L. The major limitation of this method is the requirement of light, which is not possible in large-scale production [66]. Although a high yield of resveratrol can be achieved by chemical synthesis, the production steps are complex, and the production of byproducts and the required toxic organic solvents are major drawbacks for large-scale preparations [67].

Microorganisms have been essential sources for producing pharmaceutically and industrially important compounds for decades due to low-cost cultures, fast production, the ability to construct purification processes, the ease of manipulating their genetic components, as well as the availability of protein and metabolic engineering tools [68]. Yeasts provide a more suitable platform for resveratrol production compared with bacterial hosts. As eukaryotic organisms, yeast species have the ability to express genes coding for plant-derived enzymes, such as cytochrome P450 enzymes; to perform post-translational modification (such as glycosylation); and to functionally fold the eukaryotic recombinant protein [69,70]. Additionally, yeasts and plants share similar intracellular compartments such as the endoplasmic reticulum, which supports eukaryotic and membrane proteins biosynthesis [71]. Another advantage of using yeasts is that *Saccharomyces cerevisiae* (the most-used yeast species for resveratrol production) is a food-grade organism that can be used safely in human nutrition and pharmaceutical products [72].

*S.cerevisiae* is a well-studied microorganism model for industrial and pharmaceutical applications. Metabolic engineering of *S. cerevisiae* was recognized as a robust strategy to produce several plant-derived chemicals [73]. *Yarrowia lipolytica* is another yeast that has received industrial interest for more than 50 years due to its organic acids production ability. *Y. lipolytica* is an oleaginous, non-pathogenic yeast that can accumulate lipids up to 40% of its dry cell weight (DCW) and is considered a model organism in diverse research areas [74,75]. Similar to *S. cerevisiae*, *Y. lipolytica* is classified as a generally recognized as safe organism (GRAS); therefore, it has the potential to be used in food and pharmaceutical industries. They are also considered powerful hosts for expressing heterologous genes [70,76]; consequently, both are strong candidates for use as cell factories for resveratrol production. Since microorganisms, including yeasts, cannot naturally produce resveratrol, genetic engineering of these strains by heterologous genes is required. Selecting genes to be transferred and the appropriate enzymes were found to markedly affect resveratrol production. Furthermore, pathway engineering, mutagenesis, codon optimization, protein engineering, and using synthetic scaffolds are all different strategies used to enhance resveratrol production capabilities in yeasts. Table 1 lists the genes used to engineer some yeast strains for resveratrol production.

### 3.1. Pathway Engineering

The introduction of an entire biosynthetic pathway into the microorganism provides the ability to produce resveratrol from its precursors (l-phenylalanine or l-tyrosine) or low-cost materials such as glycerol, glucose, or ethanol [19,89,98]. In this method, PAL or TAL, depending on the used pathway; C4H; 4CL; and STS encoding genes are transferred into the chosen host. One of the first attempts to entirely reconstruct the resveratrol pathway was conducted by Zhang et al. in 2006 in *S. cerevisiae*. TAL from *Rhodobacter* *sphaeroides*, in 4CL from *Arabidopsis thaliana*, and in STS from *V.* *vinifera* (4CL::STS fusion protein) were introduced into the *S. cerevisiae* strain WAT11. Although the TAL gene was not expressed in the yeast, after 20 h, 5.25 µg/mL of resveratrol was detected. The expression of the coupled genes coding for the fusion protein increased the resveratrol titer up to 15-fold compared with the co-expression of the genes encoding the separate enzymes, which emphasized the importance of the fusion protein and the spatial localization of these two related enzymes to improve resveratrol production [79]. Similarly, the same strain carrying TAL codon-optimized from *R. sphaeroides* and a similar fusion enzyme 4CL::STS were able to produce a resveratrol titer of 1.06 mg/L without the addition of l-tyrosine, and 1.90 mg/L with tyrosine. Again, the fused protein significantly increased resveratrol biosynthesis [83].

Production of resveratrol by the phenylalanine pathway was reported in *S. cerevisiae* YPH499. A strain harboring PAL from *Populus* *trichocarpa*, C4H and 4CL from *Glycine* *max,* and STS from *V. vinifera* produced resveratrol after being fed with phenylalanine, but the titer was still low (0.29 mg/L) [80]. The combination of both pathways was established in *Y. lipolytica*, and the final titer obtained was 1.46 mg/L [92]. In a recent study, *Y. lipolytica* was engineered using TAL from *Flavobacterium johnsoniae*, PAL and STS from *V.* *vinifera*, and C4H and 4CL1 from *A. thaliana*. The strains showed the ability to produce resveratrol using both pathways separately (using tyrosine or phenylalanine as precursors) or in combination. The importance of gene overexpression by increasing the gene copy number in the resveratrol pathway was clearly demonstrated in this study, in which strains harboring two copies of the PAL, C4H, 4CL1, and STS genes or two copies of TAL, 4CL1, and STS exhibited higher performances than single-copy sets of genes. The best results were obtained from a strain containing two copies of PAL, C4H, TAL, 4CL1, and STS, which produced a high titer of resveratrol, reaching 450 mg/L under fermentation conditions using 100 g/L glycerol as the sole carbon source, which is the highest reported amount of resveratrol produced from the expression of only the resveratrol pathway [94]. 

Engineering partial pathways or selected genes is an alternative strategy to produce resveratrol. In this case, PAL, TAL, and C4H are usually excluded from the pathway construction, and *para*-coumaric acid is used as the precursor. *S. cerevisiae* FY23 was the first used for resveratrol pathway construction in yeast. The 4CL216 from a hybrid poplar and vst1 from a grape vine were constitutively expressed in the strain. After feeding with 5 mM *p*-coumaric acid, the recombinant strain produced 1.45 µg/L resveratrol [77]. Higher resveratrol titers were then obtained from *S. cerevisiae* strains CEN-PK113-3B and EC1118, which harbor Nt4CL2 and At4CL2, respectively, and VvSTS. The two strains using *p*-coumaric acid as a precursor produced 5.8 and 8.2 mg/L resveratrol, respectively, as a final product [78,87].

Using synthetic scaffolds is another strategy used for improving resveratrol production. Nine different constructions containing GTPase binding domain (GBD), Src homology 3 domain (SH3) with 4CL1 from *A. thaliana*, and PSD95/DlgA/Zo-1 domain (PDZ) with STS from *V. vinifera* were recruited and optimized in *S. cerevisiae* WAT11 cells. Resveratrol production in the transformed yeast cells containing the optimal scaffold (GBD_1_SH3_2_PDZ_4_) showed a five-fold increase in the production after 36 h (6.7 mg/L) and more than a two-fold rise in the resveratrol titer at 96 h after induction (14.4 mg/L). Using this scaffold strategy increased the resveratrol titer by 2.7-fold compared with the fusion enzyme strategy for the same genes [79,85]. This indicates the effectiveness of protein scaffolds in improving resveratrol synthesis and in increasing pathway enzyme activity compared with the protein fusions strategy. 

The importance of selecting pathway genes and codon-optimizing them was explored in a recent report, in which six STS genes from different sources (PhStS, PcPKS5, MaSTS3, RtSTS, VvVST1, and AhSTS) were codon-optimized and then co-expressed with 4CL from *Plagiochasma* *appendiculatum* in *S. cerevisiae* W303. Differences in production time for the final yield were observed among the different strains. Although the lines expressing VvVST1, AhSTS, and RtSTS genes produced resveratrol quickly, their final titer was rather low (27–30 mg/L). Conversely, strains harboring STS from *Morusalba* and *P. cuspidatum* accumulated resveratrol up to 39 mg/L using 70 mg/L *p*-coumaric acid [86]. Codon optimization for resveratrol pathway genes has also been observed in several studies in both *S. cerevisiae* and *Y. lipolytica* [88,91,93,95,96]. The highest resveratrol titer, which was produced by the expression of partial pathway genes (4CL and STS) in yeasts, was obtained from industrial Brazilian sugar-cane-fermenting yeast, and the titer was 391 mg/L of resveratrol [81]. 

### 3.2. Host Metabolic Engineering (Non-Pathway Genes)

Metabolic engineering of microbial hosts for resveratrol production has achieved significant progress in recent years. The main remaining obstacles for microbial production of resveratrol are the precursor availability and the low activity of stilbene synthase in heterologous hosts. Hence, the primary strategies for increasing productivity using microbial cell factories are increasing the precursor supply (aromatic amino acids and malonyl-CoA) via genetic manipulation of the strain and improving the activity of key enzymes via protein engineering. 

**Figure 3:**
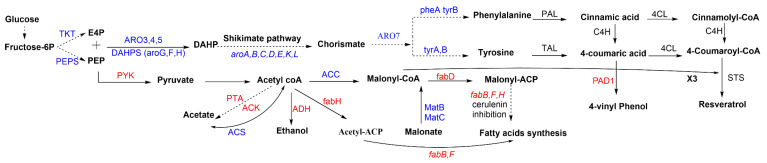
Genes and enzymes involved in the resveratrol synthesis pathway. Dotted arrows refer to multiple steps. Genes and enzymes in blue are targets for overexpression. Genes and enzymes in red are targets for knockout or inhibition. 4CL: 4-coumaroyl-coA ligase, ACC: acetyl-CoA carboxylase, Acetyl-CoA: acetyl-coenzyme A, Acetyl-ACP: acetyl-acyl carrier protein, ACS: acetyl-CoA synthase, ACK: acetate kinase, ADH: alcohol dehydrogenases, ARO3/ARO4/ARO5: 3-deoxy- d-arabinoheptulosonate-7-phosphate (DAHP) synthase, ARO7: chorismate mutase, *aroA*: gene that encodes the 3-phospho-shikimate-1-carboxyvinyltransferase protein, *aroB*: gene that encodes the dehydroquinate synthase protein, *aroC*: gene that encodes the chorismate synthase protein, *aroD*: gene that encodes the dehydroquinate dehydratase protein, *aroE*: gene that encodes the shikimate dehydrogenase protein, *aroG/aroF/aroH*: genes that encode the DAHP synthase, *aroK/aroL*: genes that encode the shikimate kinase isoenzymes I/II, C4H: cinnamate 4-hydroxylase, DAHP: 3-deoxy-d arabinoheptulosonate 7-phosphate, DAHPS: 3-deoxy-d arabinoheptulosonate 7-phosphate (DAHP) synthase, E4P: erythrose 4-phosphate, *fabB/fabF*: genes that encode the beta-ketoacyl-acp synthase I/II protein, *fabD*: gene that encodes the malonyl-CoA-acyl carrier protein transacylase, *fab*H: gene that encodes 3-oxoacyl carrier protein synthase III, fbr: feedback resistant, Malonyl-CoA: malonyl-coenzyme A, Malonyl-ACP: malonyl-acyl carrier protein, MatB: malonyl-CoA synthetase, MatC: malonate carrier protein, PAD: phenyl acrylic acid decarboxylase, PAL: phenylalanine ammonia lyase, PEP: phosphoenolpyruvate, PEPS: phosphoenolpyruvate synthase, PTA: phosphate acetyltransferase, PYK, pyruvate kinase, STS: stilbene synthase, TAL: tyrosine ammonia-lyase, TKT: transketolase, tyrA/pheA: genes that encode the chorismate mutase protein, tyrB: gene that encodes the tyrosine aminotransferase.

To increase the precursor levels, the introduction of non-pathway exogenous genes and pathway redirection are crucial. Notable efforts have been dedicated to optimizing the aromatic amino acids production and their derived phenylpropenoic acids in yeast [99,100]. Overproduction of aromatic amino acids can be easily achieved by engineering the shikimate pathway. Metabolic engineering of this pathway focused on improving carbon flux toward chorismate, and then Phe and Tyr (Figure 3). Extending the E4P and PEP supply and availability are considered fundamental approaches to enhancing chorismate production [73]. Intracellular malonyl-CoA is the other prime precursor involved in resveratrol biosynthesis. Naturally, the majority of malonyl-CoA is used in fatty acid biosynthesis, leaving a minimal level to be used in resveratrol biosynthesis. Thus, increasing this precursor in microbial hosts provides the opportunity for extra enhancement in resveratrol biosynthesis in the host platform.

Two main strategies are usually applied to enhance the malonyl-CoA pool. The first is to improve acetyl-CoA carboxylation into malonyl-CoA via the acetyl-CoA carboxylase (ACC) enzyme. The other strategy is to inhibit malonyl-CoA consumption by repressing the biosynthesis of fatty acids (Figure 3) [101]. Since the blocking of malonyl-CoA consumption and direct knockouts of the *fab* genes were found to be lethal to microorganisms [102], the inhibition has been accomplished in bacterial hosts via three procedures: the addition of cerulenin antibiotic to inhibit FabB and FabF [103,104]; using antisense RNA to repress the *fab* operon, especially the *fabD* genes [102,105]; and the CRISPRi tool, considered the third promising approach for repressing *fab* genes and directing carbon flux to malonyl-CoA [106,107]. Although these approaches have only been implemented in *E. coli*, they might provide valuable tools for future studies on increasing the precursors for resveratrol in yeast strains. *S. cerevisiae* is known to metabolize *p*-coumaric acid into 4-vinylphenol by phenyl acrylic acid decarboxylase (Pad1p). Although a PAD1 knockout mutation in *S. cerevisiae* W303-1A repressed the consumption of *p*-coumaric acid compared with the wild-type strain that uses about 60% of the *p*-coumaric acid, no enhancement was detected in resveratrol production (3.1 mg/L) when the PAD1 deletion mutant strain was transformed with a plasmid harboring At4CL1 and AhSTS [82]. In a separate study, the same strain (without PAD1 knockout mutation) was further transferred with RtPAL and AtC4H genes to produce 2.6 mg/L *p*-coumaric acid and 3.3 mg/L resveratrol, which indicates the minor effect of this strategy in increasing resveratrol production. The ACC1 gene was then overexpressed to increase the malonyl-CoA pool, which was reflected in the increase in resveratrol production to 4.3 mg/L without amino acids addition, and up to 5.8 mg/L with tyrosine added [84]. The overexpression of ACC1 was similarly reported in an *E. coli-S. cerevisiae* co-culture and *Y. lipolytica* for resveratrol production [91,93]. An increasing malonyl-CoA pool was also achieved by the overexpression of a plant malonyl-CoA synthetase (AAE13), resulting in a 2.4-fold increase and accumulation in resveratrol in *S. cerevisiae* [86].

Increasing the phenylalanine precursor in the prephenic acid pathway was achieved by overexpression of feedback-insensitive alleles encoding DAHP synthase (ScARO4^K229L^) and chorismate mutase (ScARO7^G141S^). Applying this strategy increased resveratrol production from 2.73 to 4.85 mg/L in *S. cerevisiae* with HaTAL, At4CL1, At4CL2, and VvVST1. The overexpression of the ScACC1^S659A, S1157A^ gene raised the resveratrol titer to 6.39 mg/L, and another improvement was produced by the multiple-copies integration of pathways genes, which produced the highest titer of 235.57 mg/L, being 36-fold higher than in the last strain. Eventually, 415.65 and 531.41 mg/L of resveratrol were produced from the final strain under fed-batch fermentation with glucose or ethanol as the carbon source, respectively [88]. The same previous strategies were used with the phenylalanine pathway (AtPAL2, AtC4H, At4CL2, and VvVST1) with further metabolic engineering. Overexpression of cytochrome P450 reductase (AtATR2), *S. cerevisiae* cytochrome B5 (CYB5), and acetyl-CoA synthase (SeACS^L641P^) was used to increase the precursor supply, as well the deletion of phenylpyruvate decarboxylase (ARO10) to eliminate phenylalanine competing pathways. After this extensive engineering and cultivation in a fed-batch reactor, the strain was able to produce 812 and 755 mg/L of resveratrol from glucose and ethanol, respectively [89]. 

*Y. lipolytica* was proven to be a highly promising yeast for producing resveratrol. In *Y. lipolytica* ST6512, several metabolic strategies were used to finally obtain a strain that produced 12.4 g/L resveratrol, the highest reported resveratrol titer to date from de novo production. In addition to multiple integrations of resveratrol pathway genes (*FjTAL, At4CL1*, and *VvVST1*), feedback-insensitive *YlARO4^K221L^* and *YlARO7^G139S^* were employed to enhance resveratrol production. Further improvement was achieved by optimizing the growth medium and fed-batch fermentation [96]. 

In bacterial hosts, increased precursor amounts were additionally achieved by overexpression of different genes, such as malonyl-CoA synthetase (matB), malonate carrier protein (matC), tktA, PEP synthetase (ppsA), and acetate assimilation enzyme (acs), or by deleting or inhibiting competing pathway genes such as the *pta*, *ackA, adhE, tyrR, trpR,* and *fab* genes. These strategies might enhance resveratrol production, which requires further experiments on yeast platforms [73,103,104,108,109,110,111].

## 4. Conclusions

The increased demand for resveratrol for pharmaceutical and cosmetic uses necessitates its production from sustainable sources. Yeast cells are advantageous platforms for resveratrol production because of their fast production, the ease of their cultivation and purification processes, and their remarkable potential for large-scale resveratrol production. Due to their nature as eukaryotic cells and as GRAS organisms, the *S. cerevisiae* and *Y. lipolytica* platforms are preferable for resveratrol production. The successful design of a resveratrol production pathway depends on several factors, including determining the appropriate host, examining alternative specific enzymes from various sources, optimizing the codons to maximize the expression of heterologous genes, selecting a convenient transfer gene system (plasmids and their associated genetic elements), and determining favorable culturing parameters. Metabolic engineering has also achieved significant progress in increasing the resveratrol precursors, an important limitation in resveratrol production. Despite these efforts and extensive strain engineering, heterologous stilbene synthase activity and the overall resveratrol production are relatively still low. Except for one study that produced 12 g/L of resveratrol from *Y. lipolytica* [96], almost all engineered yeast strains do not produce more than 1 g/L, which does not meet the industrial needs and is the main drawback for their use on an industrial scale. Applying all of these elements together in a balanced way as well as testing other strategies that have been applied in *E. coli* may allow scientists to obtain the desired well-designed resveratrol-producing strain. 

## Figures and Tables

**Table 1:** Production of resveratrol in different yeast hosts in different engineered yeast strains, used genes and their sources, used genetic systems for engineering, precursors, the titers obtained, and the applied scale.

Yeast/Parent Strain	Pathway Genes (Source)	Pathway/Host Engineering	Genetic System	Precursor	Titer (mg/L)	Scale	Year of Publication	Reference
*S. cerevisiae* FY23	4CL216 *(P. trichocarpa × P. deltoides*) VTS1 (*V. vinifera*)	-	Episomal plasmid	*p*-Coumaric acid	0.00145	Flask	2003	[77]
*S. cerevisiae* CEN-PK113-3B	4CL2 (*N. tabacum*) STS (*V. vinifera*)	-	One copy genome integration	*p*-Coumaric acid	5.8	Flask	2006	[78]
*S. cerevisiae* WAT11	TAL (*R. sphaeroides*) 4CL (*A. thaliana*)::STS (*V. vinifera*)	-	Episomal plasmid	*p*-Coumaric acid	5.25	Flask	2006	[79]
*S. cerevisiae* YPH499	PAL, CPRa (*P. trichocarpa × P. deltoides*) C4H, 4CL (*G. max*) STS (*V. vinifera*)	-	Episomal plasmid	Phenylalanine *p*-Coumaric acid	0.29 0.31	Flask	2009	[80]
Industrial Brazilian yeast *(S. cerevisiae*)	4CL1 (*A. thaliana*) STS (*V. vinifera*)	-	Episomal plasmid	*p*-Coumaric acid	262–391	Flask	2010	[81]
*S. cerevisiae* W303-1A	4CL1 (*A. thaliana*) STS (*A. hypogaea*)	PAD1 knockout	Episomal plasmid	*p*-Coumaric acid	3.1	Flask	2011	[82]
*S. cerevisiae* WAT11	TAL (*R. sphaeroides*) 4CL::STS, 4CL1 (*A. thaliana*)-STS *(V. vinifera*) fusion enzyme	Expression of araE transporter (*E. coli*)	One copy genome integration	Tyrosine, *p*-Coumaric acid Grape Juice	3.1 2.3 3.44	Shake flask	2011	[83]
*S. cerevisiae* W303-1A	PAL (*R. toruloides*) C4H, 4CL1 *(A. thaliana*) STS (*A. hypogaea*)	Overexpression of ACC1	Episomal plasmid	Tyrosine	5.8	Batch bioreactor	2012	[84]
*S. cerevisiae* WAT11	4CL1 (*A. thaliana*) STS (*V. vinifera*)	Synthetic scaffold	Episomal plasmid	*p*-Coumaric acid	14.4	Flask	2012	[85]
*S. cerevisiae* WAT11	4CL::STS, 4CL1 (*A. thaliana*)-STS *(V. vinifera*) fusion enzyme	Overexpression of: AAE13	One copy genome integration	*p*-Coumaric acid	Up to 3.7	Flask	2014	[86]
*S. cerevisiae* EC1118	4CL (*A. thaliana*) STS (*V. vinifera*)	-	Episomal plasmids	*p*-coumaric acid	8.249	Flask	2015	[87]
*S. cerevisiae* CEN. PK102-5B	TAL (*H. aurantiacus*) TAL (*F. johnsoniae*) 4CL1 and 4CL2 (*A. thaliana*) RS (*V. vinifera*)	Overexpression of ARO4^fbr^, ARO7^fbr^, and ACC1	Multiple copy genome integration	Glucose Ethanol	415.65 531.41	Fed-batch bioreactor Fed-batch bioreactor	2015	[88]
*S. cerevisiae* CEN. PK102-5B	PAL2, C4H, 4CL2 (*A. thaliana*) VST1 (*V. vinifera*)	Overexpression of ARO4^fbr^, ARO7^fbr^, ACC1, CYB5 (*S. cerevisiae*), ATR2 (*A. thaliana*), ACS (*S. enterica*), and deletion of aro10	Multiple-copy genome integration	Glucose Ethanol	812 755	Fed-batch bioreactor Fed-batch bioreactor	2016	[89]
*S. cerevisiae* W303	4CL1 (*P. appendiculatum*) STS (*P. henryana*) STS (*P. cuspidatum*) STS (*M. alba var. atropurpurea)* STS (*R. tataricum*) STS (*V. vinifera*) STS (*A. hypogaea*) One STS gene for each yeast line	-	Episomal plasmids	*p*-Coumaric acid	23.7–39.9	Batch bioreactor	2020	[90]
Co-culture of *E. coli* NEB10β *and* *S. cerevisiae* BY4741	TAL (*T. cutaneum*)	Overexpression of aroG and tyrA in a tyrR knockout strain	Bacterial Expression Vectors	Glucose	36	Co-culture fermentation	2020	[91]
	4CL (*A. thaliana*) STS (*V. vinifera*)	Overexpression of: ACC1	One copy genome integration	*p*-Coumaric acid (secreted from *E. coli*)				
*Y. lipolytica* ATCC 20362	PAL/TAL (*R.glutinis*) 4CL (*S. coelicolor*) STS (*V. vinifera*)	-		l-tyrosine	1.46		2010	[92]
*Y. lipolytica*	4CL (*N. tabacum*) STS (A. hypogaea)	Overexpression of: ACC1, PEX10	Randomly genome integration	*p*-Coumaric acid	48.7	Flask	2020	[93]
*Y. lipolytica* Po1d (wt), derived from W29	TAL (*F. johnsoniae*) PAL (*V. vinifera*) C4H, 4CL1 (*A. thaliana*) VST (*V. vinifera*)	-	Multiple copy genome integration	Glycerol	430	Bioreactor	2020	[94]
*Y. lipolytica* Po1fk derived from W29	TAL (*R. toruloides*) 4CL (*P. crispum*) STS (*V. vinifera*)	ARO4^fbr^ (*S. cerevisiae*) aroG^fbr^ (*E. coli*) xfpK (*B. breve*) xpkA (*A. capsulatum*) Overexpression: of ARO1, ARO2, ARO3, ARO4, ARO5, TKT Deletion of: TRP2, TRP3, ARO8, ARO9, PYK, PHA2	One copy genome integration	Glucose	12.67	Flask	2020	[95]
*Y. lipolytica* ST6512 (W29)	TAL (*F. johnsoniae*) 4CL1(*A. thaliana*) VST1 (*V. vinifera*)	Overexpression of: ARO4^fbr^ and ARO7^fbr^	Multiple copy genome integration	Glucose Glucose	409 12355	Flask Fed-batch bioreactor	2020	[96]
*Ogataea polymorpha*	TAL (*H. aurantiacus*) 4CL (*A. thaliana*) STS (*V. vinifera*)	-	CRISPR–Cas9-assisted multiplex genome editing, multi-copy integration	Tyrosine	97.23	Flask	2018	[97]

## Abbreviations

4CL	4-coumaroyl-coA ligase
AAE13	malonyl-CoA synthetase
Acetyl-CoA	acetyl-coenzyme A
Acetyl-ACP	acetyl-acyl carrier protein
ACC	acetyl-CoA carboxylase
ACS	acetyl-CoA synthase
ACK	acetate kinase
ADH	alcohol dehydrogenases
araE	arabinose transporter
ARO1	multifunctional AROM complex
ARO2	chorismate synthase
ARO3/ARO4/ARO5	3-deoxy- d-arabinoheptulosonate-7-phosphate (DAHP) synthase
ARO7	chorismate mutase
ARO8	aromatic amino acid aminotransferase I
ARO9	aromatic amino acid aminotransferase II
ARO10	transaminated amino acid decarboxylase
*aroA*	gene that encodes the 3-phospho-shikimate-1-carboxyvinyltransferase protein
*aroB*	gene that encodes the dehydroquinate synthase protein
*aroC*	gene that encodes the chorismate synthase protein
*aroD*	gene that encodes the dehydroquinate dehydratase protein
*aroE*	gene that encodes the shikimate dehydrogenase protein
*aroG/aroF/aroH*	genes that encode the DAHP synthase
*aroK/aroL*	genes that encode the shikimate kinase isoenzymes I/II
ATR2	NADPH-cytochrome P450 reductase 2
C4H	cinnamate 4-hydroxylase
CPR	cytochrome P450 reductase
CYB5	cytochrome b5
DAHP	3-deoxy-d arabinoheptulosonate 7-phosphate
DAHPS	3-deoxy-d arabinoheptulosonate 7-phosphate (DAHP) synthase
E4P	erythrose 4-phosphate
*fabB/fabF*	genes that encode the beta-ketoacyl-acp synthase I/II protein
*fabD*	gene that encodes the malonyl-CoA-acyl carrier protein transacylase
*fabH*	gene that encodes 3-oxoacyl carrier protein synthase III
fbr	feedback resistant
GRAS	generally recognized as safe
Malonyl-CoA	malonyl-coenzyme A
Malonyl-ACP	malonyl-acyl carrier protein
MatB	malonyl-CoA synthetase
MatC	malonate carrier protein
PAD	phenyl acrylic acid decarboxylase
PAL	phenylalanine ammonia lyase
PEP	phosphoenolpyruvate
PEPS	phosphoenolpyruvate synthase
PEX10	peroxisomal biogenesis factor 10
PHA2	prephenate dehydratase
PPP	pentose phosphate pathway
PTA	phosphate acetyltransferase
PYK	pyruvate kinase
STS	stilbene synthase
TAL	tyrosine ammonia-lyase
TKT	transketolase
TRP2	anthranilate synthase
TRP3	indole-3-glycerol-phosphate synthase
tyrA/pheA	genes that encode the chorismate mutase protein
TyrA	chorismate mutase/prephenate dehydrogenase
tyrB	gene that encodes the tyrosine aminotransferase
TyrR	transcriptional regulatory protein
VST/RS	resveratrol synthase
xfpK/xpkA	phosphoketolase.
